# Virological and Clinical Outcomes of Influenza Outpatients Treated With Baloxavir, Oseltamivir, or Laninamivir in the 2023–2024 Season

**DOI:** 10.1111/irv.70042

**Published:** 2024-11-18

**Authors:** Takeyuki Goto, Naoki Kawai, Takuma Bando, Yoshio Takasaki, Shizuo Shindo, Tomonori Sato, Naoki Tani, Yong Chong, Hideyuki Ikematsu

**Affiliations:** ^1^ Medicine and Biosystemic Science Kyushu University Graduate School of Medical Sciences (The First Department of Internal Medicine) Fukuoka Japan; ^2^ Japan Physicians Association Tokyo Japan; ^3^ Kawai Clinic Gifu Japan; ^4^ Bando Clinic Ishikawa Japan; ^5^ Takasaki Pediatrics and Child Health Clinic Fukuoka Japan; ^6^ Shindo Children's Clinic Fukuoka Japan; ^7^ Department of Infectious Diseases Fukuoka City Hospital Fukuoka Japan; ^8^ Department of Clinical Immunology, Rheumatology, and Infectious Disease Kyushu University Hospital Fukuoka Japan; ^9^ Ricerca Clinica co. Fukuoka Japan

**Keywords:** B Victoria, baloxavir, influenza, laninamivir, oseltamivir, variant

## Abstract

**Background:**

Clinical data on patients infected with influenza B Victoria (BV) after the approval of baloxavir is lacking.

**Methods:**

This observational study of the Japanese 2023–2024 influenza season analyzed data from 25 outpatients with A(H1N1)pdm09, 36 with A(H3N2), and 65 with BV. Viral samples were collected before and after administering an antiviral (70 patients received baloxavir and 56 received a neuraminidase inhibitor), on days 1, 5, and 10. Isolated viruses after culturing were amplified using RT‐PCR and sequenced to detect mutations of concern, including acidic protein (PA)‐amino acid (AA) E23X/I38X for influenza A and M34X/I38X for BV. Fever and symptoms were tracked via self‐reporting diaries.

**Results:**

No PA‐AA‐substituted virus was detected from 126 pre‐treatment samples. In the baloxavir cohort, one (7.1%, 1/14) PA I38F‐substituted A(H1N1)pdm09 and two (11.1%, 2/18) PA I38T‐substituted A(H3N2) viruses were isolated on day 5 but not on day 10. No (0%, 0/37) PA‐AA‐substituted BV was detected on day 5 or after. The virus isolation rate on day 5 was higher among patients with BV than with influenza A in both baloxavir (35.1% vs. 14.3% for A(H1N1)pdm09 and 16.7% for A(H3N2)) and oseltamivir‐treated patients (44.4% vs. 0% for A(H1N1)pdm09 and 33.3% for A(H3N2)). Patients with PA‐AA‐substituted influenza A after baloxavir administration did not have longer fever duration than those without virus isolation or with wild‐type virus on day 5, for both A(H1N1)pdm09 and A(H3N2).

**Conclusions:**

Baloxavir‐resistant variants were not detected in influenza BV before treatment, as with A. The emergence of PA‐AA‐substituted influenza A after baloxavir administration was temporal and did not cause prolonged symptoms. No baloxavir‐resistant BV variants were observed after baloxavir administration.

## 1 Introduction

The 2023–2024 influenza season in Japan saw the circulation of three influenza types/subtypes, A(H1N1)pdm09, A(H3N2), and B Victoria (BV), following several seasons of a significant reduction in influenza infections due to the SARS‐CoV‐2 pandemic [1]. Baloxavir marboxil (baloxavir), a selective inhibitor of the influenza polymerase acidic protein (PA) cap‐dependent endonuclease, received regulatory approval in both Japan and the United States in 2018. Phase II and III trials demonstrated that baloxavir has comparable clinical efficacy to oseltamivir and exhibits more rapid viral clearance [2, 3]. However, the emergence of PA amino acid (AA)‐substituted variants after baloxavir administration, principally I38X and E23X, which exhibit reduced susceptibility to baloxavir, has been reported [2, 3, 4].

Our previous study of the A(H1N1)pdm09 subtype during the Japanese 2019–2020 season reported a lower isolation rate for baloxavir‐treated patients compared to oseltamivir‐treated patients in both the early (day 5) and late (day 10) treatment phases (5.2% vs. 30.3%, 0% vs. 6.1%, respectively) [5]. Our subsequent study on the A(H3N2) subtype during the Japanese 2022–2023 season also reported a significantly lower isolation rate for the baloxavir‐treated patients compared to patients treated with oseltamivir and other neuraminidase (NA) inhibitors (NAIs) at both day 5 (11.1% vs. 60.0% and 52.9%, *p* < 0.001) and day 10 (0% vs. 16.7% and 6.3%, *p* = 0.0031) of treatment [6]. In both studies, PA I38X‐substituted variants were isolated (3.4% for A(H1N1)pdm09 and 5.6% for A(H3N2)) in the early phase after baloxavir treatment; however, these variants, which were expected to have reduced sensitivity to baloxavir, were not detected in the late phase, and no patients showed prolonged fever or symptom duration. These studies suggest shortened viral shedding by baloxavir and indicate that the clinical impact of the variants of concern is currently limited for influenza A. In contrast, for influenza B, the efficiency of baloxavir and the possible emergence of variants after treatment have yet to be fully described in clinical settings.

Oseltamivir, the most used NAI in Japan, has been reported to shorten the illness duration by 29 to 35 h for patients with A(H3N2) [7]. Another study showed that oseltamivir was less effective in patients with influenza B than influenza A, reporting higher virus isolation rates and longer fever durations after administration [8]. Laninamivir octanoate hydrate (laninamivir) is a long‐acting NAI that requires only a single inhalation that was approved in 2010. Our continuous surveillance has found that laninamivir‐treated patients infected with BV showed a longer fever duration than those infected with influenza A [9]. This prospective observational study was done to determine if the findings of previous seasons can be applied to the influenza A seen in the 2023–2024 season and to add new information on influenza B.

## 2 Material and Methods

### 2.1 Patients

This investigator‐initiated study was conducted across 14 collaborating institutions in Japan. Participants were recruited from outpatients of all ages who visited these clinics from December 2023 to March 2024 and were confirmed to be infected with influenza by rapid diagnostic tests. Informed consent was given by all participants or their parents if the patient was a minor. The study was approved by the Institutional Review Board of Hara‐doi Hospital (Approval Date: September 19, 2023) and was registered at the University Hospital Medical Information Network‐Clinical Trials Registry (registration no. UMIN000055124).

### 2.2 Study Procedures

Nasopharyngeal swabs or self‐collected nasal discharge for viral isolation were obtained before the initiation of antiviral treatment (day 1) and subsequently on days 5 and 10 of treatment, with some flexibility allowed. The choice of antiviral agent was at the discretion of the attending physician. All enrolled patients were administered either baloxavir or one of the NAIs: oseltamivir, laninamivir, or zanamivir in accordance with the manufacturer's instructions. Baseline clinical symptoms were recorded upon patient enrollment, as previously described [6]. The axillary temperature and seven other illness‐related symptoms (headache, muscle/joint pain, fatigue, chills/sweating, nasal congestion, sore throat, and cough) were documented via self‐reported diaries. The duration of fever was the time interval from the initiation of antiviral treatment to the attainment of an afebrile state, defined as a sustained axillary temperature below 37.5 °C. Similarly, the duration of other symptoms was determined as the time interval from the commencement of antiviral therapy to the amelioration of all recorded symptoms to a mild grade or none.

### 2.3 Influenza Virus Isolation and Typing

Clinical specimens were subjected to viral culture using Madin‐Darby Canine Kidney‐SIAT1 (MDCK‐SIAT1) cells, following previously reported protocols [10]. Viral isolation was subsequently assessed by observing the cytopathic effects in the cultured cells. For the initial samples collected on day 1, the influenza viral type and subtype were identified via reverse transcription polymerase chain reaction (RT‐PCR). Specifically, viral RNA was extracted from the culture supernatants, and RT‐PCR was performed using type‐specific and subtype‐specific primer sets, as described in previous studies [11, 12].

### 2.4 Detection of PA and NA AA Substitutions in Viral Isolates

RT‐PCR and Sanger sequencing were employed to detect the presence of E23X and I38X substitutions in the PA gene of the isolated A(H1N1)pdm09 and A(H3N2) influenza viruses, as in our previous studies [5, 6]. For influenza B, Sanger sequencing was employed to detect the presence of M34X and I38X substitutions in the PA gene with the forward primer (designated 1F) having the sequence 5′‐GGTGCGTTTGATTTGTC‐3′ and the reverse primer (designated 421R) having the sequence 5′‐GTTCCATGCTATTTCCC‐3′. RT‐PCR and Sanger sequencing were also done to identify common oseltamivir‐resistant NA mutations, specifically H275Y and N295S in the A(H1N1)pdm09, and E119X, R292K, and N294S in the A(H3N2) NA, as in our previous studies [5, 6].

### 2.5 Statistical Analyses

Statistical analyses were conducted to evaluate the significance of observed differences as follows: Fisher's exact test was employed to compare percentages across two or three distinct groups. Analysis of variance (ANOVA) was applied to parametric data among three groups. For non‐parametric data, the Wilcoxon rank sum test was used for comparing two groups, and the Kruskal‐Wallis rank sum test was applied for comparing three groups. A *p*‐value of < 0.05 was considered statistically significant. All statistical computations were executed using R software (version 4.1.1) in conjunction with R Studio (version 2021.09.0).

## 3 Results

### 3.1 Patient Characteristics

Of the 130 participants who initially met the enrollment criteria, four were removed from the dataset due to the inability to isolate virus from their day 1 samples. The baseline clinical characteristics of the final cohort of 126 patients, stratified by subtype, are shown in Table 1. Twenty‐five patients were confirmed to be infected with influenza A(H1N1)pdm09, 36 with A(H3N2), and 65 with B Victoria (BV). Among the three subtypes, the mean age was significantly lower for patients with BV than for other patients (12.4 years vs. 32.4 years for A(H1N1)pdm09 and 22.0 years for A(H3N2), *p* < 0.001). The history of influenza infection in the previous (2022–2023) season was significantly lower for patients with A(H3N2) than for other patients (5.6% vs. 20.0% for A(H1N1)pdm09 and 27.7% for BV, *p* = 0.019). Patients with BV had the lowest influenza vaccination rate in the 2023–2024 season (13.8% vs. 40.0% for A(H1N1)pdm09 and 25.0% for A(H3N2), *p* = 0.029). The prevalence of underlying disease was highest for patients with A(H1N1)pdm09 (36.0% vs. 8.3% for A(H3N2) and 7.7% for BV, *p* = 0.002). There was no significant difference among the three subtypes in initial body temperature, symptom score, or choice of antiviral treatment. Seventy patients (14 with A(H1N1)pdm09, 19 with A(H3N2), and 37 with influenza B virus) were treated with baloxavir. Fifty‐six patients (11 with A(H1N1)pdm09, 17 with A(H3N2), and 28 with influenza B virus) were treated with one of the NAIs. No significant difference was observed among the three subtypes on the actual sampling days corresponding to the scheduled collection days 5 and 10 (Table S1).

**TABLE 1:** Baseline clinical characteristics stratified by virus subtype.

	*n*		Virus type/subtype			*p*‐Value
		Total	A(H1N1)pdm09	A(H3N2)	B Victoria	
		126	25	36	65	
Sex	Male, *n* (%)	69 (54.8)	15 (60.0)	25 (69.4)	29 (44.6)	0.049^a^
	Female, *n* (%)	57 (45.2)	10 (40.0)	11 (30.6)	36 (55.4)	
Age	< 12 years, *n* (%)	61 (48.4)	11 (44.0)	13 (36.1)	37 (56.9)	0.120^a^
	≥ 12 years, *n* (%)	65 (51.6)	14 (56.0)	23 (63.9)	28 (43.1)	
	Range, yrs	1–89	2–89	1–72	1–61	
	Mean ± SD, yrs	19.1 ± 20.5	32.4 ± 30.6	22.0 ± 22.1	12.4 ± 8.9	< 0.001^b^
Influenza in the previous season	Yes, *n* (%)	25 (19.8)	5 (20.0)	2 (5.6)	18 (27.7)	0.019^a^
Influenza vaccination in 2023–24 season	Yes, *n* (%)	28 (22.2)	10 (40.0)	9 (25.0)	9 (13.8)	0.029^a^
Underlying disease	Yes, *n* (%)	17 (13.5)	9 (36.0)	3 (8.3)	5 (7.7)	0.002^a^
Immunocompromised host	Yes, *n* (%)	0 (0)	0 (0)	0 (0)	0 (0)	
Body temperature at initial visit						
Total	Mean ± SD, °C	38.3 ± 0.9	38.3 ± 0.9	38.2 ± 0.9	38.3 ± 0.9	0.852^b^
< 12 years	Mean ± SD, °C	38.4 ± 0.9	38.4 ± 0.9	38.3 ± 1.0	38.5 ± 0.9	0.895^b^
≥ 12 years	Mean ± SD, °C	38.1 ± 0.9	38.2 ± 0.8	38.1 ± 0.9	38.1 ± 0.9	0.984^b^
Influenza symptom score at initial visit						
Total	Mean ± SD	7.3 ± 3.8	6.9 ± 4.3	7.9 ± 3.6	7.1 ± 3.7	0.510^b^
< 12 years	Mean ± SD	6.1 ± 3.2	5.8 ± 3.1	6.1 ± 3.2	6.2 ± 3.3	0.937^b^
≥ 12 years	Mean ± SD	8.4 ± 4.0	7.7 ± 5.0	8.9 ± 3.5	8.2 ± 4.0	0.671^b^
Antiviral treatment						
Baloxavir	Yes, *n* (%)	70 (55.6)	14 (56.0)	19 (52.8)	37 (56.9)	0.971^a^
NAIs	Yes, *n* (%)	56 (44.4)	11 (44.0)	17 (47.2)	28 (43.1)	0.971^a^
Oseltamivir	Yes, *n* (%)	24 (19.0)	6 (24.0)	9 (25.0)	9 (13.8)	0.295^a^
Laninamivir	Yes, *n* (%)	28 (22.2)	3 (12.0)	7 (19.4)	18 (27.7)	0.270^a^
Zanamivir	Yes, *n* (%)	4 (3.2)	2 (8.0)	1 (2.8)	1 (1.5)	0.314^a^

Abbreviation: NAIs, neuraminidase inhibitors.

^a^ Fisher's exact test.

^b^ Analysis of variance (ANOVA).

### 3.2 Detection of PA and NA AA‐Substitutions

No virus with the PA AA‐substitution (E23X/I38X for influenza A and M34X/I38X for BV) was identified in the 126 pre‐treatment (day 1) samples across all treatment groups and viral subtypes (data not shown). The prevalence of specific variants associated with resistance to baloxavir (E23X/I38X for influenza A and M34X/I38X for BV) and to the NAIs (NA H275Y/N295S for A(H1N1)pdm09 and E119X/R292X/N294X for A(H3N2)) in the post‐treatment period is presented in Table 2. In the day 5 samples from the patients treated with baloxavir (baloxavir group), PA I38X‐substitutions were observed in 7.1% (one I38F out of 14) for A(H1N1)pdm09 and 11.1% (two I38T out of 18) for A(H3N2). No BV variant with PA M34X/I38X‐substitution was detected. No PA or NA variant was detected across the three subtypes in day 5 and 10 samples from the patients treated with an NAI (NAI group).

**TABLE 2:** Post‐treatment detection of viruses in influenza patients with baloxavir or other treatment in the 2023–2024 season in Japan.

Antiviral treatment	Virus type/subtype									*p* ^a^ for % Isolated
	A(H1N1)pdm09			A(H3N2)			B Victoria			
	No. tested	No. Isolated (%)	No. variants (%)^b^	No. tested	No. Isolated(%)	No. variants (%)^c^	No. tested	No. Isolated(%)	No. variants (%)^d^	
Baloxavir										
Early post‐treatment phase (day 5)^e^	14	2 (14.3)	1 (7.1)	18	3 (16.7)	2 (11.1)	37	13 (35.1)	0 (0)	0.218
Late post‐treatment phase (day 10)^e^	13	0 (0)	0 (0)	18	0 (0)	0 (0)	36	0 (0)	0 (0)	—
NAIs										
Early post‐treatment phase (day 5)^e^	11	2 (18.2)	0 (0)	17	6 (35.3)	0 (0)	28	7 (25.0)	0 (0)	0.673
Late post‐treatment phase (day 10)^e^	11	0 (0)	0 (0)	17	1 (5.9)	0 (0)	28	0 (0)	0 (0)	0.500
Oseltamivir										
Early post‐treatment phase (day 5)^e^	6	0 (0)	0 (0)	9	3 (33.3)	0 (0)	9	4 (44.4)	0 (0)	0.198
Late post‐treatment phase (day 10)^e^	6	0 (0)	0 (0)	9	1 (11.1)	0 (0)	9	0 (0)	0 (0)	>0.999
Laninamivir										
Early post‐treatment phase (day 5)^e^	3	1 (33.3)	0 (0)	7	2 (28.6)	0 (0)	18	3 (16.6)	0 (0)	0.500
Late post‐treatment phase (day 10)^e^	3	0 (0)	0 (0)	7	0 (0)	0 (0)	18	0 (0)	0 (0)	>0.999

Abbreviations: NA, neuraminidase; NAIs, neuraminidase inhibitors; PA, polymerase acidic protein.

^a^ Fisher's exact test.

^b^ The detection of PA E23X/I38X variants for day 1, and PA E23X/I38X or NA H275Y/N295S variants for days 5, 10.

^c^ The detection of PA E23X/I38X variants for day 1, and PA E23X/I38X or NA E119X/R292X/N294X variants for days 5, 10.

^d^ The detection of PA M34X/I38X variants for days 1, 5,and 10.

^e^ The first and second samples after treatment were collected on the scheduled days 5 and 10, respectively.

### 3.3 Viral Isolation After Antiviral Treatment for Influenza

The rates at which residual influenza viruses were isolated in the post‐treatment period are shown in Table 2. For the baloxavir group, BV showed a higher isolation rate (13/37, 35.1%) than did A(H1N1)pdm09 (2/14, 14.3%) and A(H3N2) (3/18, 16.7%) at day 5. No virus was isolated from the baloxavir group at day 10. For the NAI group, A(H3N2) showed the highest isolation rate at day 5 (6/17, 35.3%), compared to 18.2% (2/11) for A(H1N1)pdm09 and 25.0% (7/28) for BV. The breakdown of the NAI group is as follows: for patients treated with oseltamivir (oseltamivir group), the day 5 isolation rates were 0% (0/6) for A(H1N1)pdm09, 33.3% (3/9) for A(H3N2), and 44.4% (4/9) for BV; for patients treated with laninamivir (laninamivir group), the day 5 isolation rates were 33.3% (1/3) for A(H1N1)pdm09, 28.6% (2/7) for A(H3N2), and 16.6% (3/18) for BV. For the oseltamivir group, only one sample had virus isolated at day 10, A(H3N2). No statistically significant difference was observed in the isolation rates of the three subtypes regardless of antiviral treatment.

### 3.4 Duration of Fever

The durations of fever for patients, stratified by antiviral treatment and viral subtype, are shown in Table 3. In the baloxavir group, patients infected with BV did not exhibit a significant difference in fever duration (median 24.0 h, IQR 18.5–32.5) when compared to the other subtypes (median 18.5 h, IQR 13.0–25.0 for A(H1N1)pdm09 and median 23.0 h, IQR 21.0–37.0 for A(H3N2), *p* = 0.223). In the oseltamivir group, patients infected with BV showed a significantly longer fever duration (median 58.5 h, IQR 38.8–84.5) than the other subtypes (median 18.0 h, IQR 14.2–21.0 for A(H1N1)pdm09 and median 31.0 h, IQR 15.0–43.0 for A(H3N2), *p* = 0.029). In the laninamivir group, patients with BV did not show a significant difference in fever duration (median 34.0 h, IQR 19.0–50.5) when compared to those with the other subtypes (median 56.0 h, IQR 50.0–62.0 for A(H1N1)pdm09 and median 32.0 h, IQR 20.0–41.0 for A(H3N2), *p* = 0.528). Patients with PA‐AA‐substituted influenza A after baloxavir administration did not have a longer fever duration than those without virus isolation or those with wild‐type virus on day 5, for both A(H1N1)pdm09 (median 10.0 h vs. 18.5 h and 36.0 h, *p* = 0.165) and A(H3N2) (median 20.5 h vs. 24.0 h and 37.0 h, *p* = 0.447). Among the patients with BV in the oseltamivir group, patients with virus isolation positive in day 5 samples showed a significantly longer fever duration than did those negative, with a median of 86.0 h versus 37.5 h (*p* = 0.028). This difference was not observed in the baloxavir and laninamivir groups.

**TABLE 3:** Duration of fever during the 2023–2024 influenza season in Japan.

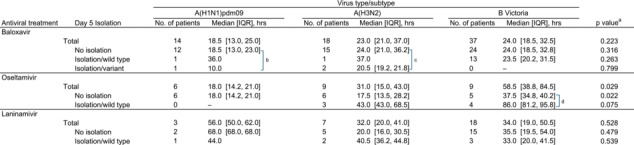

^a^ Kruskal–Wallis rank sum test for three groups and Wilcoxon rank sum test for two groups.

^b^ *p* = 0.165, Kruskal–Wallis rank sum test.

^c^ *p* = 0.447, Kruskal–Wallis rank sum test.

^d^ *p* = 0.028, Wilcoxon rank sum test.

### 3.5 Duration of Influenza Related Symptoms

The durations of influenza‐related symptoms, stratified by antiviral treatment and viral subtype, are shown in Table 4. In the baloxavir group, patients infected with BV did not show a significant difference in symptom duration (median 104.0 h, IQR 47.5–144.0) when compared to those with the other subtypes (median 70.5 h, IQR 53.0–132.0 for A(H1N1)pdm09 and 64.0 h, IQR 48.0–118.0 for A(H3N2), *p* = 0.640). In the oseltamivir group, patients with BV did not exhibit a significant difference in symptom duration (median 144.0 h, IQR 94.5–144.0) when compared to those with the other subtypes (median 49.0 h, IQR 47.0–66.0 for A(H1N1)pdm09 and median 73.0 h, IQR 49.0–95.0 for A(H3N2), *p* = 0.636). In the laninamivir group, patients with BV did not show a significant difference in symptom duration (median 88.0 h, IQR 64.0–118.0) when compared to those with the other subtypes (median 144.0 h, IQR 108.5–144.0 for A(H1N1)pdm09 and 95.5 h, IQR 90.8–98.0 for A(H3N2), *p* = 0.133). Patients with PA‐AA‐substituted influenza A after baloxavir administration did not show a significant difference in symptom duration when compared to those without virus isolation or those with wild‐type virus on day 5, for both A(H1N1)pdm09 (median 10.0 h vs. 18.5 h and 36.0 h, *p* = 0.444) and A(H3N2) (median 20.5 h vs. 24.0 h and 37.0 h, *p* = 0.524). Among the patients with BV in the oseltamivir group, those with virus isolated in day 5 samples showed a longer symptom duration than those without isolation, with a median of 144.0 h versus 70.0 h (*p* = 0.242). This difference was not observed in the baloxavir and laninamivir groups.

**TABLE 4:** The duration of influenza‐related symptoms during the 2023–2024 influenza season In Japan.

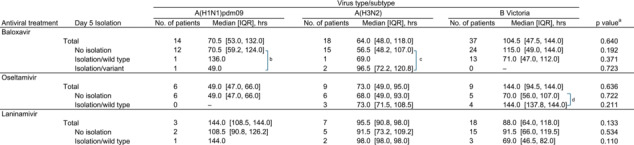

^a^ Kruskal–Wallis rank sum test for three groups and Wilcoxon rank sum test for two groups.

^b^ *p* = 0.444, Kruskal–Wallis rank sum test.

^c^ *p* = 0.524, Kruskal–Wallis rank sum test.

^d^ *p* = 0.242, Wilcoxon rank sum test.

## 4 Discussion

Unlike the 2022–2023 season, in which A(H3N2) activity predominated, the A(H1N1)pdm09, A(H3N2), and B Victoria types/subtypes were all observed in the 2023–2024 season in Japan [1]. The baseline characteristics of our patients, presented in Table 1, show that those infected with BV in this study were significantly younger than those infected with influenza A, consistent with previous observations [9, 13, 14]. As for A(H3N2), potential protection from immunity after A(H3N2) infection in the previous season may have contributed to the low frequency of past infection in this cohort.

The spread of baloxavir‐resistant variants with PA‐AA substitutions into the community is of concern. To address this, we continued monitoring influenza viruses with PA‐AA substitutions in pre‐treatment samples, as we have done over the past several seasons. No PA‐AA‐substituted viruses were isolated in 91 A(H1N1)pdm09 samples in the pre‐treatment period during the 2019–2020 season or from 73 A(H3N2) samples in the 2022–2023 season [5, 6]. In this study, among 126 collected samples, including 65 B Victoria subtype samples, no PA‐AA‐substituted viruses were isolated in the pre‐treatment period. These results align with the report from the national surveillance of influenza antiviral drug resistance in Japan, which shows a low frequency of baloxavir resistance: 0.3% for A(H1N1)pdm09, 0.7% for A(H3N2), and 0% for BV [15]. To date, the variant of concern has not shown signs of accumulating in the community for any of the three influenza types/subtypes, even in Japan, where baloxavir use is relatively high.

In a review of in vitro studies [16], PA I38X‐substituted virus showed 2.5‐ to 49.5‐fold, 5.6‐ to 614.0‐fold, and <3‐fold changes in effective concentrations (EC_50_) for A(H1N1)pdm09, A(H3N2), and BV, respectively. In that report, PA E23X‐substituted A(H1N1)pdm09 showed a 6.9‐ to 7.4‐fold change, and PA M34X‐substituted BV showed a <3‐fold change in EC_50_. In this study, PA E23X/I38X‐substituted viruses were isolated in 7.1% (1/14) of A(H1N1)pdm09 and 11.1% (2/18) of A(H3N2) samples in the early post‐treatment period, a slight difference compared to our previous reports of 3.4% (2/58) for A(H1N1)pdm09 and 5.6% (2/36) for A(H3N2) [5, 6], likely due to the small number of influenza A cases analyzed this season. As in the previous studies, the variants detected in the early post‐treatment period were not persistently isolated in the late post‐treatment period, and they seemed not to affect the duration of fever or symptoms as compared to the wild type (Tables 3 and 4). For BV, none of the 37 samples from the baloxavir group had PA M34X/I38X‐substituted virus in the early post‐treatment period. Given the low inhibitory effect in vitro and the extremely low detection rate of variant strains in clinical settings, concerns about PA‐AA‐substituted viruses after baloxavir treatment appear to be less significant for influenza BV than for influenza A.

The overall percentage of BV isolated in the early post‐treatment period in our baloxavir group was more than twice that of the other two subtypes, despite no potential baloxavir‐resistant BV variant being detected. Supplemental data from a previous study reported that the median time to sustained cessation of infectious virus detection among baloxavir‐treated high‐risk patients was 24.0 h (95% CI: 24.0–48.0 h) for A(H3N2) and 72.0 h (95% CI: 48.0–96.0 h) for BV [3]. Baloxavir may not be as efficient at clearing influence B virus as it is in clearing influenza A virus.

Regarding NA, a recent review of antiviral‐resistant substitutions reported that no substitutions were detected in BV that exhibited a highly reduced inhibition by oseltamivir or laninamivir. In contrast, NA H275Y and N295S substitutions in A(H1N1)pdm09 exhibited highly reduced inhibition by oseltamivir [16]. NA E119X, R292X, and N294X substitutions in A(H3N2) have also been reported to significantly increase resistance to oseltamivir [17, 18]. In our analysis, none of the aforementioned NA substitutions were detected in the virus samples isolated on or after day 5.

We investigated the duration of fever and symptoms to evaluate differences in virus types/subtypes and antiviral treatments. Patients harboring the PAI38X‐substituted virus did not show a longer fever duration than those with the wildtype virus, consistent with our previous observations [5, 6]. In the oseltamivir group, patients infected with BV had longer fever durations than those infected with A(H1N1)pdm09 and A(H3N2). Specifically, patients infected with BV whose virus was isolated on day 5 exhibited longer fever durations than those whose virus was not isolated. A higher virus isolation rate after oseltamivir treatment and a longer fever duration for patients with influenza B than for those with influenza A have been previously reported [8, 19]. The longer viral shedding with oseltamivir against BV might be related to prolonged fever. Our previous study on laninamivir‐treated patients, which analyzed eight seasons, reported that patients with BV had a longer fever duration compared to those with influenza A [9]. In this study, no significant differences were observed, likely due to the small number of laninamivir‐treated patients for each type/subtype. When comparing the treatment groups, the fever and symptom durations for BV tended to be shorter in the baloxavir than in the oseltamivir group. Another study of children also reported that baloxavir more effectively shortened the fever duration of patients with influenza BV than did an NAI [20]. The virological difference between influenza A and B might have more impact on the anti‐viral effectiveness of NAIs than PA cap‐dependent endonuclease inhibitor.

Another limitation of this study, in addition to the small sample size, is that we used viral isolates for RT‐PCR and sequencing, consistent with the methodology of our prior studies. Therefore, the isolation rates in this study cannot be directly compared with the detection rates of clinical specimens in other studies. Nevertheless, we were able to observe three types of influenza during the 2023–2024 season and add valuable information on BV, which has been limited since the approval of baloxavir. The impact of the PA‐AA‐substituted virus in BV is limited in clinical settings, consistent with findings for A(H1N1)pdm09 and A(H3N2) in our previous series of studies [5, 6].

## Author Contributions

**Takeyuki Goto:** formal analysis, writing – original draft, data curation, software, visualization. **Naoki Kawai:** conceptualization, resources, investigation. **Takuma Bando:** conceptualization, investigation, resources. **Yoshio Takasaki:** investigation, resources. **Shizuo Shindo:** investigation, resources. **Tomonori Sato:** data curation, formal analysis, validation. **Naoki Tani:** validation, writing – review and editing. **Yong Chong:** writing – review and editing, methodology. **Hideyuki Ikematsu:** supervision, conceptualization, methodology, writing – review and editing, project administration.

## Conflicts of Interest

H. Ikematsu has previously received honoraria from Shionogi & Co., Ltd., and Daiichi Sankyo Co., Ltd. for medical advice and lectures.

### Peer Review

The peer review history for this article is available at https://www.webofscience.com/api/gateway/wos/peer‐review/10.1111/irv.70042.

## Supporting information


**Table S1** The actual sampling point for each scheduled day.
